# The Development and Preliminary Validation of a Brief Questionnaire of Psychopathic Personality Traits

**DOI:** 10.3389/fpsyg.2017.01471

**Published:** 2017-09-05

**Authors:** Sonja Etzler, Sonja Rohrmann

**Affiliations:** ^1^Department of Psychology, Goethe University Frankfurt Frankfurt, Germany; ^2^Centre for Criminology Wiesbaden, Germany

**Keywords:** psychopathy, personality questionnaire, FPP, factorial invariance, test validity

## Abstract

The measurement of psychopathic personality traits via self-report has become an important tool in legal psychology. One prominent instrument is the Psychopathic Personality Inventory (PPI; Lilienfeld and Andrews, 1996), a well-validated questionnaire that is widely applied in many countries. In Germany, it is the only questionnaire assessing psychopathic traits that is available from a publisher with a manual edited for easy administration. Nevertheless, the PPI shows certain shortcomings: the high number of 154 items makes it less economic, it was developed on a non-representative undergraduate sample, and studies revealed an inconsistent factor structure. To overcome these points, a new questionnaire, the Questionnaire of Psychopathic Personality Traits [German: Fragebogen Psychopathischer Persönlichkeitseigenschaften (FPP)] was developed. The sample consists of *n* = 132 civilians (56% female) and *n* = 173 inmates of German correctional facilities (30% female). The FPP comprises 30 items, whose wording was short and adequate for inmates. It shows satisfying psychometric properties regarding factorial structure, item properties, and reliability. Partial invariance regarding both subsamples allows for interpretation of latent means. Results supported validity such as associations with self-reported crime, and inmates’ misconduct. The factorial structure was cross-validated on a second sample of *N* = 517 participants (71% female) from an online study. The FPP is useful in large-scale research studies as well as for clinical settings, e.g., for treatment planning in correctional facilities.

## Introduction

The psychopathic personality disorder has been an important construct in clinical and legal psychology because it is thought to be associated with antisocial and criminal behavior. Thus, it plays an important role in risk assessment as well as treatment planning in legal settings (Hemphill et al., 1998). Diagnostic tools for assessment of psychopathy are established such as the Psychopathic Checklist-Revised (PCL-R; Hare, 2003) or more recently the Comprehensive Assessment of Psychopathic Personality (CAPP; Cooke et al., 2012) both relying on records and clinical interview. Since 20 years also self-report assessment has been considered as assessment method and several studies suggest that it can be a promising alternative to external ratings, especially under anonymous or pseudonymous conditions (Ray et al., 2013). In their study, Miller et al. (2011) compared self and informant reports on psychopathic traits in a community sample and revealed that convergence was strong for total scores of psychopathy (*r* = 0.67^∗∗^) assessed via Psychopathic Personality Inventory-Revised (PPI-R; Lilienfeld and Widows, 2005). Self-report assessment of psychopathy has additional advantages (Lilienfeld, 1994). It uses participants’ insight to obtain diagnostically relevant information. Furthermore, economy and standardization of the test administration play a crucial role in large-scale studies, where time and financial resources are limited. Finally, a multi-method approach could help to separate the variance of traits and methods and lead to a better understanding of psychopathy (Skeem and Cooke, 2010). There are several questionnaires for measuring psychopathy available, most of them developed in North America (Hare, 1985; Levenson et al., 1995; Lilienfeld and Andrews, 1996; Patrick et al., 2009). These instruments differ in terms of the definition of psychopathy they are based on, in the degree of validation and in adaption to other cultures and languages.

The Psychopathic Personality Inventory (PPI; Lilienfeld and Andrews, 1996; Lilienfeld and Widows, 2005)^1^ is based on a relatively broad approach to psychopathy. Since today, the PPI in its original and its revised version is probably the best established questionnaire for assessing psychopathy and has been translated into many languages (e.g., German, French, Spanish). Until now it is the only questionnaire for psychopathic traits in Germany that is available from a publisher with a manual edited for easy administration. A number of different studies has tested its validity and reliability (for an overview see Miller and Lynam, 2011). Nevertheless, this inventory has been subject to some criticism.

The PPI comprises a large number of items (187 and 154, respectively), so that it is not sufficiently economic: completing the questionnaire takes 30–45 min. This expenditure of time causes high costs and may bias the sample. Since completing the questionnaire demands high efforts, the willingness to participate in a study may depend on certain personality traits that are associated with psychopathy itself (e.g., the ability to concentrate, openness to experience, and conscientiousness etc.).

The wording of some of the PPI items is long and complex, therefore comprehension and interpretation might vary between subjects. This may affect comparative studies analyzing differences between civil and inmate samples, especially if inmates have lower educational level. Second, some items are inadequate for inmates, because they refer to aspects that may be difficult to evaluate in prison (e.g., “The opposite sex finds me sexy and appealing.” or “I don’t care about following the rules”). This reduces the applicability of the questionnaire and violates the criterion of reasonableness of items. Some items are highly situation-specific (e.g., “It might be exciting to be on airplane that was about to crash but somehow landed safely.”). In their Item Response Theory analyses Cooke et al. (2006) found that items of the PCL-R show the highest diagnostic information if they are formulated in a general manner. This could be explained with the idea that psychopathy as an “open concept” (Cronbach and Meehl, 1955) is associated with the probability of certain kinds of behavior and does not consist of an exhaustive number of specific behaviors. Increased situational specificity may decrease the discriminatory power of some items. But, however, highly general formulated items also require high inferential ability.

Self-report instruments for psychopathy tend to be saturated with the higher-order trait of negative affectivity. As Arias-Martín (2011) demonstrated, the subscales of the PPI, *Blame Externalization* and *Stress Immunity* in particular, show high correlations with depression. If a measure is heavily saturated with negative affectivity is has diminished specificity regarding psychopathy, because high levels of negative affectivity are found in a variety of psychiatric conditions (Lilienfeld, 1994).

To define the structure of psychopathy, the authors of the PPI included all facets of psychopathy that were subject of any definitions in the scientific literature and converted them into items. The facets of the PPI were obtained through analyzing all items with principal component analyses (PCA) and assign them to one of the eight orthogonal components. Generally speaking, PCA is based on the correlative structure of all items, and since correlation indices are not able to separate core features of psychopathy from mere associated characteristics, the validity of the superordinate factor PPI-I Fearless Dominance was strongly questioned because it failed to show strong correlations with measures of constructs related to empathy, aggression, substance abuse or antisocial behavior (Miller and Lynam, 2011). Although Lilienfeld et al. (2012) emphasized the importance of Fearless Dominance within the framework of psychopathy as Cleckley’s *Mask of Sanity*, Lynam and Miller (2012) pointed out that for reasons of validity its correlation with antisocial behavior should reach a substantial effect size. This stresses the importance of Fearless Dominance in the conception of psychopathy as well as the importance of its association with antisocial behavior.

The PCA was based on a non-criminal sample of undergraduates. But correlation indices are conditioned by variances that limited variances might reduce covariance (Cohen et al., 2003). If psychopathic traits and criminal behavior correlate, choosing only non-criminal psychopaths lead to a reduction of psychopathic trait variance. In their study, Uzieblo et al. (2007) reported means and variances for a subsample of male undergraduates (*N* = 167) and for inmates (*N* = 165). Comparing both variances it results that the PPI-scores vary significantly stronger in inmates than in male undergraduates, the same kind of sample, the PPI was developed on. Although the authors assume that psychopathic traits vary within the civil population, strictly speaking, the obtained scales (facets) and their structure reflect only adaptive psychopathy, as it is manifested in a non-criminal sample rather than a comprehensive conception of the construct.

### Development of the Questionnaire of Psychopathic Personality Traits (FPP)

Based on the criticism concerning the facets of psychopathy captured by the PPI, the present study started with a review process in order to assemble all symptoms that previously have been confirmed as core traits of psychopathy in qualitative, theoretical, and empirical approaches. We started with the eight facets that are captured by the PPI subscales and their conceptualization of the psychopathic personality (Lilienfeld and Andrews, 1996). Based on other influential definitions of psychopathy (e.g., Cleckley, 1941; McCord and McCord, 1964; Levenson, 1992; Hare, 2003), and in order to define facets with content that resemble constructs already defined in differential psychology, we changed the eight subscales in a simpler and empirically updated model.

We retained the facet Fearlessness since it has been found as core trait in several definitions of psychopathy (Cleckley, 1941; Lykken, 1995; Lilienfeld and Andrews, 1996). Empathy is a construct whose characteristics have been broadly studied (Davis, 1996; Jolliffe and Farrington, 2004; Decety and Ickes, 2011), and many studies suggest that only the facet emotional empathy, not perspective taking is impaired in psychopaths (Blair, 2008; Jones et al., 2010). Therefore, we included the facet Lack of (emotional) Empathy instead of PPI-Coldheartedness. We further changed PPI-Impulsive Nonconformity into Impulsivity since many studies emphasize the core role of impulsivity within psychopathy (Hart and Dempster, 1997; Hare, 2003) and, other than PPI-Impulsive Nonconformity, its conceptualization as well as its nomological net is widely examined in differential psychology (e.g., Webster and Jackson, 1997). Short term aspects of PPI-Carefree Nonplanfulness are partly reflected in the facet Impulsivity, as long term aspects are confounded with age and were therefore excluded for the following conceptualization. In order to emphasize antisocial aspects of social interaction (Babiak and Hare, 2006), PPI-Social Potency was changed into Social Manipulation and later on, Power was included to catch the aspect of dominating others (Benning et al., 2003). PPI-Machiavellian Egocentricity was changed into Narcissistic Egocentricity to emphasize narcissistic feelings of grandiosity, as implied in the PCL-R as item 2 *Grandiose Sense of Self Worth* (Hare, 2003). PPI-Stress Immunity was excluded because it was partly reflected by components of Fearlessness. PPI-Blame Externalization was excluded because it turned out to be a reverse pattern of depression (Arias-Martín, 2011) and was therefore assumed not to be specific indicator for psychopathy (for a discussion of this topic see Lilienfeld, 1994).

After this review the six concepts of *Lack of Empathy, Fearlessness, Narcissistic Egocentrism, Impulsivity, Social Manipulation*, and *Power* are assumed to constitute the second-order dimensional trait *Psychopathy*.

*Lack of Empathy* is regarded as an important core feature of psychopathy (Cleckley, 1941; Lilienfeld and Andrews, 1996; Hare, 2003), i.e., a lack of compassion when perceiving strong negative emotions like fear, depression, or rage in others (Brook and Kosson, 2013). This deficit is assumed to concern only emotional empathy, the ability to *feel* the same emotional state as the communication partner (Preston and de Waal, 2002), but not perspective taking, i.e., the ability to *comprehend* her or his situation.

Another core symptom (Cleckley, 1941; Karpman, 1948; Fowles, 1980; Lykken, 1995) is *Fearlessness*, a low physiological sensitivity for and poor ability of anticipating negative consequences of one’s own conduct. Accordingly, psychopaths show low behavioral inhibition, even in the face of negative consequences (Hall, 2009). In his *low fear hypothesis*, Lykken (1995) evaluates fearlessness as central in the pathogenesis of psychopathy: a genetic disorder causes a dysfunction in fear conditioning, so that the normal process of socialization fails to create the mechanism of conscience which usually constrains antisocial impulses and a psychopathic phenotype emerges.

Another important symptom is *Narcissistic Egocentrism*, the belief that everyone needs to look after himself or herself (“survival of the fittest”), combined with a feeling of superiority over others. Cleckley (1941) observes that psychopaths are exceedingly self-centered, which can become visible to different degrees. Thus, psychopaths are absorbed by their own needs and ignore those of other people (McCord and McCord, 1964). Combined with their feeling of superiority, this triggers behaviors serving subjects own interests and harming others at the same time.

Another often discussed facet of psychopathy is *Impulsivity*, which means that a person responds to internal and external stimuli spontaneously, without self-control and often with inappropriate behaviors. McCord and McCord (1964) characterize psychopaths as so high in impulsiveness that they have difficulties in following a daily routine or pursuing long-term goals.

*Social Manipulation* refers to high social skills that are predominantly used to manipulate others (Hare, 2003). Cleckley (1988) describes these skills as “superficial charm and good intelligence,” which are employed to reach personal goals.

*Power* is the urge to influence others and to make them or even force them to behave in a desired way. Central to this aspect is that the individual profits from others by using them as “objects” (Levenson, 1992). Some researchers call this “dominance” (PPI-I “Fearless Dominance,” Benning et al., 2003) viewed as a general facet of psychopathy.

### Aim of the Study

The present study aims at developing a new self-report instrument to measure psychopathic traits. It should further exploit the potential benefits of self-report assessment and should be usable for research and as screening instrument in practical settings. After item selection we want to evaluate the FPP in five steps. First, we test if the FPP exhibits the postulated structure with six correlated factors and in a second model, if these factors have a common second-order factor representing psychopathy. Second, psychometric properties of items and reliability of the total scale as well as the subscales will be examined. The third step will be the test of factorial invariance between the civil and the inmate sample to reveal whether meaningful interpretation of latent mean differences will be possible. Fourth, we investigate construct validity in terms of correlation indices. We expect an overall positive correlation of the PPI with FPP. Additionally, we expect high associations between counterparts and relating facets of PPI and FPP: PPI-Fearlessness with Fearlessness, PPI-Coldheartedness and Lack of Empathy, PPI-Machiavellian Egocentricity with Narcissistic Egocentricity, PPI-Social Potency with Social Manipulation and Power, PPI-Impulsive Nonconformity with Impulsivity, PPI-Carefree Nonplanfulness with Impulsivity and Fearlessness, and PPI-Stress Immunity with Fearlessness. PPI-Blame Externalization was not expected to show high association to any subscale, since we aimed to avoid saturation with depression and negative affectivity. Furthermore, we expect positive associations with narcissistic, and antisocial personality style, as well as with dominance. Negative associations are expected with avoidant and selfless personality style as well as attachment, since psychopathy is associated with emotional detachment and negative interpersonal orientation (Hare, 2003; Fowles and Dindo, 2009). No associations are expected between the PPI validity scale Unlikely Virtues. Furthermore, no association was expected with positive and negative affectivity, to ensure that the FPP is not highly saturated with these traits in order to maintain specificity toward psychopathy. In terms of criterion validity, we expect that the FPP correlates with indices of antisocial and criminal behavior, such as imprisonment and self-reported antisocial and criminal conduct. We expect inmates to show higher scores in the FPP total score as well as in all subscales. For the inmate sample, associations with criminal history, current offense and misconduct in prison are postulated.

## Materials and Methods

### Item and Scale Construction

To make the FPP suitable for non-criminals as well as offenders, the items needed to be interpretable irrespective of educational level or environmental settings. Therefore, complicated formulations and items that do not apply to prison life (e.g., questions concerning travels or sexual relationships) were avoided. Item content was to be of medium situational specificity and to ensure content validity and adequate wording. To reduce the influence of *social desirability* as well as the *opportunity to dissimulate*, item wording kept face validity and social desirability low. In the first step, items were formulated according to the facets definition and the requirements listed above. In the second step, these items were reviewed by independent psychologists specialized in the field of psychological diagnostics and test construction. In the third step the item pool was checked by experienced psychologists employed in penal institutions, and discussed with the authors with regards to item wording and content validity. The result is a preliminary test version of 60 items. Scale description and sample items can be found in **Table 1**.

**Table 1:** Scales and exemplary items of the questionnaire of psychopathic personality traits (FPP).

Description	Item examples
**(1) Lack of Empathy (inverted scale) (FPP-E)**	
People with a pronounced lack of empathy show little compassion when perceiving strong emotions in others, especially negative emotions (anxiety, depression, anger).	“It has happened that a highly emotional book/film made me cry.” “When I see a fearful face I can feel the anxiety myself.”

**(2) Fearlessness (FPP-F)**	
People high in Fearlessness have little ability to anticipate negative consequences of their own behavior and are highly insensitive to them.	“When doing something I rarely think that it might go wrong.” “There are few things that frighten me.”

**(3) Narcissistic Egocentrism (FPP-N)**	
People with high Narcissistic Egocentrism are very self-centered; this includes the belief that everybody can only look after herself or himself (survival of the fittest) as well as the idea of being superior to others and more valuable.	“Usually I cannot show consideration for others.” “I believe that I am superior to most others.”

**(4) Impulsivity (FPP-I)**	
Being highly impulsive means reacting spontaneously and without control, often with inadequate responses to internal and external stimuli.	“Others say that I easily fly off the handle.” “When I see something that I like I must have it immediately.”

**(5) Social Manipulation (FPP-S)**	
To score high in this trait, an individual has to be socially competent and use this skill for manipulating others.	“I know how to make others like me.” “When I try hard, I can make others believe everything I say.”

**(6) Power (FPP-P)**	
A high manifestation of this trait represents a strong need to influence others and to make them behave in the desired manner including the use of coercion.	“I love it when others do what I tell them to do.” “If others don’t do what I want them to do I may use physical violence.”

### Sample

The present study comprises two main samples with a total of *N* = 305 participants. The first sample consists of *n* = 132 civilians who completed the questionnaires. The second sample comprises *n* = 173 inmates of German correctional facilities who completed the questionnaires and agreed to a file review by the test administrators.

Civil participants were *M* = 35.8 (*SD* = 15.7) years old, 56% were female. Their highest educational degree was university degree (44%), university entrance, or equivalent level (22%), completed apprenticeship (22%), secondary school certificate (8%), or secondary modern school leaving certificate (2%).

Inmates were *M* = 30.8 (*SD* = 12.4) years on average, 30% of them were recruited in a women’s prison. Prison sentences ranged from few weeks to 20 years, with an average of 3 years (*SD* = 3 years). There was no selection regarding specific kinds of offenses, 38% had committed violent offenses such as robbery or assault, 5% had committed sexual offenses such as rape or child molestation. Furthermore, 19% had committed fraud, and 12% property crimes, 10% were condemned for homicide, 9% for violations of the narcotics law, 9% for others. For 56% it was the first time in prison. Their highest educational degrees were distributed as follows: 34% school leaving certificate, 29% no certificate, 16% completed apprenticeship, 15% secondary school certificate, and 3% university entrance or equivalent level. Only 2% reported a university degree.

Additionally, for the purpose of cross-validation, a third sample of *N* = 513 participants took part of an online study. Participants were *M* = 26.4 (*SD* = 7.1) years old, 71% of them were female. Their highest educational degree was university degree (48%), university entrance, or equivalent level (45%), completed apprenticeship (3%) or secondary school certificate (1%).

### Assessments

#### Questionnaire of Psychopathic Personality Traits

The test comprises 60 items in its preliminary version. Items are rated on a six-point Likert scale with *0* = *absolutely not true* to *5* = *absolutely true*.

#### Psychopathic Personality Inventory-Revised (PPI-R; Lilienfeld and Widows, 2005)

To assess psychopathy, we used the German adaption (Alpers and Eisenbarth, 2008) of the English original (Lilienfeld and Widows, 2005). It comprises eight subscales: Blame Externalization, Rebellious Nonconformity, Coldheartedness, Social Potency, Carefree Nonplanfulness, Fearlessness, Machiavellian Egocentricity, Stress Immunity. Additionally, it includes one validity scale, the Unlikely Virtues Scale. The total PPI-R comprises 154 items that are rated on a four-point Likert scale from *0 = wrong* to *3 = right*. The total PPI-R has demonstrated a good internal consistency of α = 0.93 in a German sample. Validity was supported by high correlations with the PCL-R (*r* = 0.43; Poythress et al., 2010) and institutional misconduct (*r* = 0.29; Edens et al., 2008).

#### Personality Style and Disorder Inventory (PSSI; Kuhl and Kazén, 2009)

The Personality Style and Disorder Inventory (German: Persönlichkeits-Stil- und Störungs-Inventar; PSSI) assesses personality styles that in their extreme manifestations correspond to clinical personality disorders (PDs). The PSSI scales comprises 10 items each and items were rated on a four-point Likert scale, ranging from *1 = does not apply at all*, to *4 = does apply completely*. The following scales were used: Ambitious – Narcissistic PD (α = 0.76), Self-Assertive – Antisocial PD (α = 0.85), Self-Critical – Avoidant PD (α = 0.78), and Helpful – Selfless PD (α = 0.79). Validity was for example supported by correlations of Ambitious-Narcissistic PD and the Narcissistic Personality Inventory (*r* = 0.58) and generally with expected associations of subscales with psychosomatic symptoms (Kuhl and Kazén, 2009).

#### Checklist for Antisocial Behavior and Criminality (CAV/K; Etzler et al., in press)

The CAV/K (German: Checkliste für Antisoziale Verhaltensweisen und Kriminalität; CAV/K) was administered to assess antisocial behavior and criminality via self-report. It was developed for this study and comprises 18 items measuring the frequency of antisocial behaviors. Exemplary items are: “I beat someone up so strongly that he had to go to the doctor,” or “I changed or forged a certificate (for example school certificate).” Behaviors can be rated on a six-point scale from *0 = never* to *5 = very often*. Additionally, each item receives a second criminal behavior rating, whether respondents had been convicted for this conduct or not. Ratings can be done either with *0 = no* or *1 = yes*. Internal consistency was α = 0.90 for Antisocial Behavior and α = 0.85 for Criminality. Validity of Antisocial Behavior is supported by correlations with, for instance, education *r* = -0.494, p < 0.001 and Number of violent offenses *r* = 0.222, p < 0.001. Validity of Criminality is supported by correlations with education *r* = -0.546, *p* < 0.001, number of violent offenses, *r* = 0.323, *p* < 0.001, and length of prison sentence *r* = 0.246; *p* < 0.001.

#### Positive and Negative Affect Schedule (PANAS; Watson et al., 1988)

To assess positive affect (PA) and negative affect (NA) we used the German translation (Krohne et al., 1996) of the English original (Watson et al., 1988). The Positive and Negative Affect Schedule (PANAS) assesses PA (α = 0.84) and NA (α = 0.86) with 10 items each. The PANAS can be presented either with an instruction referring to current affects (states) or an instruction referring to general affects (traits). In this study, only the general instruction assessing traits was used. Validity is reflected by correlations of vigilance (*r* = 0.27) and anxiety (*r* = 0.54) with NA and Extraversion with PA (*r* = 0.45, Krohne et al., 1996). Additionally, the expected two-factors were found in the German sample, supporting factorial validity. This questionnaire was administered to civilians only.

#### Relationship and Attachment Inventory (BB-PI; Andresen, 2012)

The Relationship and Attachment Inventory (German: Beziehungs- und Bindungs-Persönlichkeitsinventar; BB-PI) assesses traits that are important within romantic partner selection and romantic relationships as well as cohabitation. It comprises eight scales, each of them consists of 18 items. Two scales were selected to measure the interpersonal facets Dominance (18 items, α = 0.91) and Attachment (18 items, α = 0.90). Items were rated on a five-point Likert scale from *1 = absolutely wrong* to *5 = absolutely right*. Correlations between Dominance and Aggressiveness (*r* = 0.60) as well as between Attachment and the importance of relationships (*r* = 0.34) support validity. This questionnaire was administered to civilians only.

#### Hamburg Personality Inventory (HPI; Andresen, 2002)

To assess focus on norms and control, only one subscale of the Hamburg Personality Inventory (HPI) was selected. The scale Focus on Norms and Control consists of 14 items and exhibits an internal consistency of α = 0.84. Items were rated on a four-point Likert scale ranging from *1 = does not apply at all* to *4 = does apply completely*. Several studies support validity of the questionnaire (Andresen, 2002). This questionnaire was administered to civilians only.

#### Screening Instrument of Predicting Violent Offenses (SVG-5; Eher et al., 2012)

To assess the age of the first violent offense, and the number of violent offenses the Screening Instrument of Predicting Violent Offenses (German: Screeninginstrument zur Vorhersage des Gewaltrisikos; SVG-5) was applied. It contains five items that are associated with risk to recidivate of violent offenders. Interrater reliability was revealed to be high with *r*_tt_ = 0.88 for its predecessor SVG-10 and predictive validity regarding violent reoffenses was high with *AUC* = 0.79 (Rettenberger et al., 2010). This instrument was administered to inmates only.

### Ethics Statement

Ethical approval and compliance with data protection act were granted by the Hessian Ministry of Justice. The study was carried out with written informed consent from all subjects. The surveys did not collect any health information from the participants. The introduction page clearly stated that participation was voluntary and anonymous, and that participants had the right to withdraw from the study at any time. The study was conducted in agreement with the Declaration of Helsinki.

### Procedure

Participants of the civil population were recruited among contacts of the project team. Two 25 € vouchers were raffled off among the participants for reward. Inmates were recruited with posters/flyers, participation for inmates was voluntary and rewarded with an expense allowance of 5 €. Civil participants had to fill in all questionnaires listed above.

The inmate sample completed questionnaires assessing psychopathy, personality styles and antisocial and criminal behaviors only, because time was limited due to organizational reasons in prison. Seven raters were trained to fill in a standardized rating sheet to analyze files of inmates as well as the SVG-5. Variables of the current offense were type of offense and the corresponding sentence inmates were serving at the time of data collection. With regards to criminal history, we collected officially registered offenses before incarceration out of a file which was originally drawn from German Federal Central Criminal Register at the time of conviction. Furthermore, we registered whether the current incarceration was the first imprisonment of the offender and the number of probation revocations. Misconduct in prison was assessed by registering the number of educational and disciplinary measures, and the corresponding misconducts. Forms of parole were assessed, ranging from “no parole” to “conditional leave for several days.”

In a second study civil participants of the cross-validation study were recruited via Facebook to fill in the FPP and two other questionnaires not analyzed in the current study. Participation was voluntarily and was not rewarded.

### Statistical Analyses

All calculations were conducted with SPSS 22 and M*plus* version 7.1 (Muthén and Muthén, 1998–2012). Missing data for the FPP was 2.5%. With regard to confirmatory factor analyses (CFA), reliability, and factorial invariance it was treated applying Full Information Maximum Likelihood estimation (FIML; Muthén and Muthén, 1998–2012). In all other analyses missing values in FPP scales were estimated applying Expectation Maximization (EM, Lüdtke et al., 2007).

#### Confirmatory Factor Analyses (CFA)

Since preliminary analyses revealed that the distribution of indicator variables deviated from normality, a robust Maximum Likelihood estimation was chosen for CFA and for tests of factorial equivalence (Muthén and Muthén, 1998–2012). To evaluate model fit, the χ^2^-Test was supplemented by further indices, due to its high statistical power in large samples (Bollen, 1989). Following Hu and Bentler (1999), model fit was considered acceptable if χ^2^*/df* ≤ 2, *RMSEA* ≤ 0.06, *SRMR* ≤ 0.08, and *CFI* ≥ 0.95. Factor loadings of λ ≥ 0.300 were considered as acceptable.

#### Reliability

To determine reliability in terms of internal consistency, McDonald’s ω was calculated, due to its weak assumption of τ-congeneric measurement (McDonald, 1999; Zinbarg et al., 2005). This was preferred over Cronbach’s α, which presupposes the stricter essential τ-equivalence, an assumption which partially has to be rejected for this questionnaire.

#### Factorial Invariance

To test whether the factorial model applied to civilians and inmates is alike, factorial equivalence was tested stepwise in multiple group analyses, using a forward approach (Gregorich, 2006; Dimitrov, 2010). Three models were compared: first, configural invariance was tested, that factorial structure and the pattern of factor loadings are equal for both subsamples. Second, factor loadings were set equal between both groups to test metric invariance. Third, the scalar model additionally included equal intercepts for both groups. Scalar invariance is viewed as a prerequisite for interpreting latent mean differences, because it ensures the equality of source and scale unit (Moshagen et al., 2014). As the χ^2^-difference test of two nested models depends on sample size and number of observed variables, Cheung and Rensvold (2002) recommend testing for difference with the help of the difference of *CFI* and of McDonalds Non-Centrality Index. Values of *Δ_CFI_* ≤-0.01 and *Δ_NFI_* ≤-0.02 indicate a substantial deterioration of model fit, so that the hypothesis of measurement equivalence has to be rejected.

#### Zero Inflated Negative Binomial Regression

Self-reported criminality constitutes a count variable. Fitting a standard regression line to predict count criminality, the assumption of normality of residuals is violated (Atkins and Gallop, 2007). Negative binomial regression (NB-regression) is the appropriate model for data consisting of counts. Studying criminality in civilians, there is a stack of zeros in the data, because many participants have never engaged in criminal behavior. Zero-inflated NB-regression approximates the complete distribution of the outcome by two components. A logistic model is estimated for the “zero/non-zero” aspect of the outcome and an NB-regression for the counts part of the model.

## Results

### Item Selection

In order to obtain an economic questionnaire, we conducted three steps to reduce the number of items from 10 to 5. In the first step, items that turned out to have misleading or ambiguous wording were removed according to test protocols of our data collection in prison. In the second step, we conducted CFAs with one latent factor for every subscale to examine the measurement model. We identified item pairs that show high error covariances through significant modification indices indicating redundancy with regard to content validity. Items with high redundancy were removed in the second step, following a rank order of significant error covariances instead of a predefined cut-off value. In the third step, remaining items were evaluated with regard to high factor loadings in the measurement model as well as regression coefficients for the prediction of self-reported crime in the complete sample (*N* = 305) as well as criminal history and the length of prison sentence within inmates (*n* = 173). Also in this step, items were removed according to their rank within one measurement model, not following a specific cut-off value. At the end of this selection process, we maintained five items per subscale resulting in 30 items for the final version of the FPP.

### Factorial Structure

Model fit of the six-factor model with intercorrelated factors (Model 1) was adequate, except for the *CFI*, with χ^2^(390) = 750, *p* < 0.001; χ^2^*/d*f = 1.92; *RMSEA* = 0.055 [0.049–0.061]; *CFI* = 0.84; *SRMR* = 0.071. Factor loadings are printed in **Table 2**, intercorrelations of factors are shown in **Table 3**. In Model 2, a second-order factor psychopathy was considered. Indices mostly document close to acceptable model fit except for the *CFI*, χ^2^(399) = 815, *p* < 0.001; χ^2^*/df* = 2.04; *RMSEA* = 0.058 [0.053–0.064]; *CFI* = 0.81; *SRMR* = 0.081, factor loadings of first order factors are shown in **Table 3**.

**Table 2:** Item characteristics of the FPP items (*N* = 305).

Item	λ	*r_it_*	*P_(i)_*	*Var*	*M*
**Lack of Empathy (FPP-E) (i)**					
1	0.462	0.459	32	2.90	1.59
7	0.645	0.592	48	3.08	2.38
13	0.668	0.580	41	1.83	2.05
19	0.840	0.684	34	1.74	1.71
25	0.751	0.624	40	1.65	2.01
**Fearlessness (FPP-F)**					
2	0.588	0.498	49	1.86	2.47
8	0.727	0.622	53	1.96	2.63
14	0.373	0.307	48	1.65	2.40
20	0.665	0.525	44	2.56	2.21
26	0.612	0.482	42	2.13	2.08
**Narcissistic Egocentrism (FPP-N)**					
3	0.456	0.345	34	1.57	1.71
9	0.452	0.397	33	2.08	1.65
15	0.451	0.389	27	1.37	1.35
21	0.702	0.474	37	1.62	1.85
27	0.442	0.261	40	1.54	1.98
**Impulsivity (FPP-I)**					
4	0.341	0.323	47	1.89	2.33
10	0.746	0.623	34	2.30	1.69
16	0.649	0.539	54	2.15	2.69
22	0.675	0.579	50	3.15	2.48
28	0.755	0.650	41	1.87	2.03
**Social Manipulation (FPP-S)**					
5	0.709	0.596	53	1.63	2.66
11	0.355	0.352	53	1.48	2.64
17	0.624	0.563	63	1.53	3.14
23	0.718	0.583	49	1.70	2.46
29	0.637	0.500	42	1.28	2.09
**Power (FPP-P)**					
6	0.557	0.394	58	1.27	2.89
12	0.345	0.283	30	1.38	1.50
18	0.664	0.519	45	1.90	2.24
24	0.535	0.424	43	2.10	2.13
30	0.656	0.566	36	1.53	1.81

*λ = factor loadings Model 1, *r_*it*_* = item discrimination coefficient, *P_*(i)*_*= item difficulty coefficient. Scale 1, FPP-E Lack of Empathy (i), is inverted.*

**Table 3:** Characteristics of scales and factors, respectively (*N* = 305).

	ω	α	*M*	*SD*	FPP-E	FPP-F	FPP-N	FPP-I	FPP-S	FPP-P
FPP-E	0.80	0.79	9.8	5.5	1					
FPP-F	0.74	0.73	11.7	4.9	0.502^∗∗a^	1				
					0.438^∗∗b^					
FPP-N	0.63	0.62	8.6	4.0	0.692^∗∗a^	0.509^∗∗a^	1			
					0.500^∗∗b^	0.371^∗∗b^				
FPP-I	0.78	0.77	11.2	5.4	0.257^∗∗a^	0.188^∗a^	0.291^∗∗a^	1		
					0.202^∗∗b^	0.197^∗∗b^	0.221^∗∗b^			
FPP-S	0.75	0.75	13.0	4.3	0.337^∗∗a^	0.449^∗∗a^	0.783^∗∗a^	0.180^∗a^	1	
					0.280^∗∗b^	0.388^∗∗b^	0.527^∗∗b^	0.177^∗∗b^		
FPP-P	0.69	0.68	10.6	4.2	0.242^∗∗a^	0.303^∗∗a^	0.660^∗∗a^	0.406^∗∗a^	0.762^∗∗a^	1
					0.199^∗∗b^	0.239^∗∗b^	0.427^∗∗b^	0.327^∗∗b^	0.544^∗∗b^	
FPP	0.90	0.87	64.9	18.7	0.337^∗∗c^	0.298^∗∗c^	0.972^∗∗c^	0.104^∗∗c^	0.657^∗∗c^	0.507^∗∗c^

*ω = McDonalds ω. α = Cronbach’s α. FPP-E = Lack of Empathy, FPP-F = Fearlessness, FPP-N = Narcissistic Egocentrism, FPP-I = Impulsivity, FPP-S = Social Manipulation, FPP-P = Power.*

*^a^Factor intercorrelations of Model 1; ^b^manifest subscale intercorrelations; ^c^factor loadings of Model 2.*

*^∗^*p* < 0.05; ^∗∗^*p* < 0.01.*

### Characteristics of Items and Scales, Reliability

Detailed item characteristics can be found in **Table 2**. All factor loadings were higher than λ = 0.300; the same was true for most of the item discrimination indices, except for two which were smaller than *r*_it_ = 0.300 (λ_27_ = 0.261; λ_12_ = 0.283). The scales FPP-E and FPP-I had particularly high variances in comparison to FPP-N and FPP-P. Intercorrelations of factors as well as intercorrelations of manifest subscales were positive and significant (see **Table 3**); however, FPP-I tended to correlate only moderately with the other scales.

Reliability of the whole test was estimated to be ω = 0.90 of which ω = 0.73 was accounted to the total scale, and ω = 0.17 was accounted to the specific variance of subscales. Reliabilities of the subscales were located between ω = 0.63 and ω = 0.79 (**Table 3**). Reliability of two facets were not acceptable with values of FPP-N ω = 0.63 and FPP-P ω = 0.69, positioned under the critical value of ω = 0.70. The specific portion of subscales reliability was FPP-E ω = 0.04, FPP-F ω = 0.03, FPP-N ω = 0.00, FPP-I ω = 0.05, FPP-S ω = 0.02, and FPP-P ω = 0.02. Note, that this is not the total reliability of subscales rather than the additional specific true variance of each scale. To facilitate comparison with other self-report measures of psychopathy, Cronbach’s α is reported in **Table 3**, although the assumption of τ-equivalent models could not be fully met by all scales and results should be interpreted with caution.

### Factorial Invariance

Confirmatory factor analyses Model 1 was used to test for factorial invariance. **Table 4** contains fit indices for model fit and difference tests of the models. Model fit of the configural model (Model 1) was acceptable. Equalizing factor loadings between groups (Model 2) caused a small deterioration of model fit with *Δ_CFI_* = -0.008 und *Δ_NCI_* = -0.015, but relying on the criterion of *Δ_CFI_* ≤ -0.01 and *Δ_NFI_* ≤-0.02, metric invariance is achieved. Equalizing the intercepts of both groups (Model 3) in the next step caused a significant and strong deterioration of model fit, *Δ_CFI_* = -0.059 and *Δ_NCI_* = -0.093 and χ^2^(24) = 180.9, *p* < 0.001, so that scalar invariance does not apply to the data.

**Table 4:** Model fit in testing factorial invariance of inmate and civilian samples (*N* = 305).

Model	χ^2^	df	*p*	RMSEA	CFI	Reference	Δ_χ^2^_	Δ_df_	Δ_p_	Δ_CFI_	Δ_NCI_
						model					
(1) Configural	1266	780	<0.001	0.064	0.803						
(2) Metric	1310	804	<0.001	0.064	0.795	1	43.5	24	0.0087	–0.008	–0.015
(3) Scalar	1480	828	<0.001	0.072	0.736	2	180.9	24	<0.001	–0.059	–0.093
(3b) Partial scalar	1354	822	<0.001	0.065	0.785	2	44.0	18	0.0006	–0.010	–0.018

*χ^*2*^ = Robust χ^*2*^ – Statistics, *RMSEA* = root mean square error of approximation, Δ_χ^*2*^_ = corrected χ^*2*^ – difference test, Δ_*df*_ = difference of degrees of freedom, _Δ _*CFI*__ = difference of *CFI*-values, Δ_*NCI*_ = difference of *NCI*-values.*

Modification indices were used to examine the source of the lack of scalar invariance. We released equality constraints of six items in Model 3b, partial invariance. Compared to the Model 2, the partial invariance model shows no deterioration, with *Δ_CFI_* ≤-0.010 and *Δ_NFI_* ≤-0.018. Inmates showed higher intercepts than civilians independent of values in latent factors such as psychopathy and its subscales for item 7 (“Extreme violence in TV”), item 9 (“to help others”), item 4 (“to act without reflection”), item 22 (“act without control”), item 11 (“Impress others”), and item 12 (“Tolerate no dissent”). This partial invariance is sufficient to interpret latent mean differences (Byrne et al., 1989).

### Construct Validity

To test for convergent construct validity, we analyzed correlations of FPP with PPI that can be found in **Table 5**. The majority of the correlation indices are positive and significant. FPP-E is correlated with all subscales and the total scale of the PPI, showing the highest association with *Coldheartedness* as expected (*r* = 0.672, *p* < 0.001, *n* = 283). FPP-F is positively correlated with all subscales of the PPI except *Carefree Nonplanfulness* and exhibits highest correlations with *Impulsive Nonconformity* (*r* = 0.473, *p* < 0.001, *n* = 277) and its equivalent *Fearlessness* (*r* = 0.445, *p* < 0.001, *n* = 293). FPP-N is positively associated with five subscales, showing the highest association with its counterpart *Machiavellian Egocentricity* (*r* = 0.595, *p* < 0.001, *n* = 280). FPP-I shows relatively low but predominantly significant correlations with subscales of the PPI, the highest being with *Blame Externalization* (*r* = 0.380, *p* < 0.001, *n* = 281) and *Impulsive Nonconformity* (*r* = 0.337, *p* < 0.001, *n* = 277). However, it is also negatively correlated with *Stress Immunity* (*r* = -0.384, *p* < 0.001, *n* = 280). FPP-S correlates highest with *Social Potency* (*r* = 0.638, *p* < 0.001, *n* = 276) as hypothesized. FPP-P exhibits the highest correlation with *Machiavellian Egocentricity* (*r* = 0.516, *p* < 0.001, *n* = 280) followed by the association with *Social Potency* (*r* = 0.460, *p* < 0.001, *n* = 276) as expected. The scales of the PPI that do not have a direct equivalence within the FPP – *Carefree Nonplanfulness, Blame Externalization*, and *Stress Immunity* – exhibit somehow lower correlations with subscales and the total scale of the FPP, although positive and significant. Nevertheless, *Blame Externalization* is well-represented by the FPP-E (*r* = 0.311, *p* < 0.001, *n* = 281), FPP-F (*r* = 0.291, *p* < 0.001, *n* = 281), and FPP-I (*r* = 0.380, *p* < 0.001, *n* = 281) as well as the total FPP (*r* = 0.343, *p* < 0.001, *n* = 300). The total score of FPP correlates with all subscales of PPI positively and significant showing the highest association with the PPI total (*r* = 0.677, *p* < 0.001, *n* = 300). Correlations of the FPP scales and the *Unlikely Virtues Scale* indicate that there is no association with FPP total but small correlations with FPP-F (*r* = 0.238, *p* < 0.001, *n* = 279), FPP-S (*r* = -0.133, *p* = 0.026, *n* = 279), and FPP-P (*r* = -0.151, *p* = 0.012, *n* = 279).

**Table 5:** Correlations between FPP and PPI subscales (n from 276 to 300).

Scales of PPI-R	FPP-E	FPP-F	FPP-N	FPP-I	FPP-S	FPP-P	FPP
Machiavellian Egocentricity	0.335^∗∗^	0.159^∗∗^	0.595^∗∗^	0.214^∗∗^	0.520^∗∗^	0.516^∗∗^	0.559^∗∗^
Social Potency	0.183^∗∗^	0.298^∗∗^	0.271^∗∗^	0.073	0.638^∗∗^	0.460^∗∗^	0.460^∗∗^
Coldheartedness	0.672^∗∗^	0.313^∗∗^	0.447^∗∗^	0.044	0.227^∗∗^	0.164^∗∗^	0.478^∗∗^
Carefree Nonplanfulness	0.197^∗∗^	0.069	0.104	0.212^∗∗^	–0.031	–0.030	0.144^∗^
Fearlessness	0.319^∗∗^	0.445^∗∗^	0.224^∗∗^	0.121^∗^	0.219^∗∗^	0.174^∗∗^	0.381^∗∗^
Blame Externalization	0.311^∗∗^	0.291^∗∗^	0.104	0.380^∗∗^	0.112	0.079	0.343^∗∗^
Impulsive Nonconformity	0.395^∗∗^	0.473^∗∗^	0.464^∗∗^	0.337^∗∗^	0.473^∗∗^	0.381^∗∗^	0.625^∗∗^
Stress Immunity	0.189^∗∗^	0.371^∗∗^	0.103	–0.384^∗∗^	0.272^∗∗^	–0.032	0.118^∗^
PPI-total	0.532^∗∗^	0.535^∗∗^	0.488^∗∗^	0.234^∗∗^	0.543^∗∗^	0.379^∗∗^	0.677^∗∗^
Unlikely Virtues Scale	0.116	0.238^∗∗^	0.008	0.044	–0.133^∗^	–0.151^∗^	0.048

*FPP-E = Lack of Empathy, FPP-F = Fearlessness, FPP-N = Narcissistic Egocentrism, FPP-I = Impulsivity, FPP-S = Social Manipulation, FPP-P = Power.*

*^∗^*p* < 0.05; ^∗∗^*p* < 0.01.*

Further correlation analyses reveal construct validity with other traits. As expected, correlations were found with *dominance*^2^ (*r* = 0.429, *p* < 0.001, *n* = 132), *ambitious – narcissistic personality style (PS)* (*r* = 0.433, *p* < 0.001, *n* = 304), *self-critical – avoidant PS* (*r* = -0.220, *p* < 0.001, *n* = 304) and *helpful – selfless PS* (*r* = -0.307, *p* < 0.001, *n* = 304). A particularly high correlation was found with *self- assertive – antisocial PS* (*r* = 0.750, *p* < 0.001, *n* = 304). As further expected, FPP did not correlate with the PANAS scales, *positive affect^2^* (*r* = 0.067, *p* = 0.450, *n* = 131), and *negative affect^2^* (*r* = 0.094, *p* = 0.287, *n* = 131). Furthermore, there was no association between FPP and *Focus on norms and control^2^* (*r* = 0.058, *p* = 0.513, *n* = 131) and a positive association with *attachment^2^* (*r* = 0.193, *p* = 0.027, *n* = 132) both not expected in our hypotheses.

To better understand these unexpected results, we computed additional correlations. Between *Focus on norms and control* and FPP subscales (*n* = 131) were notable effect sizes with FPP-E (*r* = -0.155, *p* = 0.078), FPP-S (*r* = 0.198, *p* = 0.023), and FPP-P (*r* = 0.217, *p* = 0.013), all other coefficients being almost zero correlations. Between *attachment* and FPP subscales (*n* = 132), we found notable effect sizes with FPP-N (*r* = 0.185, *p* = 0.034), FPP-I (*r* = 0.222, *p* = 0.011), FPP-P (*r* = 0.244, *p* = 0.005), all other coefficients being zero almost correlations.

### Criterion Validity

#### General Imprisonment

**FIGURE 1:**
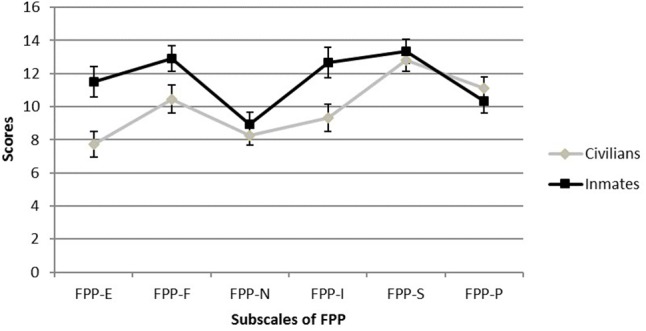
Means and standard errors of FPP subscales for inmates and civilians. FPP-E = Lack of Empathy; FPP-F = Fearlessness; FPP-N = Narcissistic Egocentrism; FPP-I = Impulsivity; FPP-S = Social Manipulation; FPP-P = Power. *N* = 305.

To examine criterion validity, we analyzed associations of FPP and its subscales with imprisonment. **Figure 1** presents subscale means for inmates and civilians separately.

Descriptively, **Figure 1** shows that 5 of the 6 means are higher for inmates than for civilians, only FPP-P is higher for civilians than for inmates. A subsequent MANOVA revealed that the factor Imprisonment was significant, with *Wilks’ Λ* = 0.766, *F*(6,298) = 15.14, *p* < 0.001. Results of *post hoc* ANOVAs yielded significant differences for 3 of 6 subscales, FPP-E *F*(1,303) = 33.7, *p* < 0.001, η^2^ = 0.100, FPP-F *F*(1,303) = 17.2, *p* < 0.001, η^2^ = 0.055, and FPP-I *F*(1,303) = 28.2, *p* < 0.001, η^2^ = 0.085. There were no significant differences depending on Imprisonment in the means of the other subscales, FPP-N *F*(1,303) = 1.1, *p* = 0.294, η^2^ = 0.004, FPP-S *F*(1,303) = 1.1, *p* = 0.304, η^2^ = 0.003, and FPP-P *F*(1,303) = 2.3, *p* = 0.126, η^2^ = 0.008. The differences between the inmate and the civilian sample remained significant when age, sex, and education were controlled for, *Wilks’ Λ* = 0.947, *F*(6,288) = 2.7, *p* = 0.016.

In the overall score of the FPP, inmates showed higher means (*M* = 68.9, *SE* = 1.60) than civilians (*M* = 59.6, *SE* = 1.18). We conducted a *t*-test and calculated an accelerated and bias-corrected bootstrapped confidence interval because this represents a more precise estimation than confidence intervals based on the assumption of normality or the bootstrapped percentile method (Efron, 1987). This difference between inmates and civilians (-9.26, BCa 95% CI [-13.2, -5.1]) was significant, *t*(296) = -4.657, *p* < 0.001, with a medium effect size, *d* = 0.68.

#### Self-Reported Antisocial and Criminal Behavior

A simple regression analysis revealed that the overall FPP score was highly predictive of antisocial behavior, with *R^2^* = 0.401, *F*(1,291) = 195, *p* < 0.001. An increase of one point in the overall FPP score caused a significant increase of *b* = 0.479 points in antisocial behavior, again considering an accelerated and bias corrected bootstrapped confidence interval BCa 95% CI [0.399, 0.548], *p* < 0.001.

To predict self-reported criminal behavior, a zero-inflated negative binomial regression was calculated with the FPP as predictor being z-standardized. Results are presented in **Table 6**. The logistic part of the model predicts the probability of belonging to the group of zeros (“not criminal”). As the overall FPP score increases one standard deviation, the probability of reporting some kind of criminal act increases by 48%. The count part of the model predicts the frequency of criminal behavior, an increase of one standard deviation in the predictor FPP is associated with a 24.5% increase of the (incidence) rate of self-reported crimes.

**Table 6:** Summary of zero inflated negative binomial regression for FPP predicting crime (*n* = 278).

Variable	*B*	*SE B*	*Z*	*p*	
	Logistic portion of the model, AV = Crim#0				Odds ratio

Intercept	–0.668	0.187	–3.572^∗∗^	<0.001	0.513
zFPP	–0.652	0.159	–4.115^∗∗^	<0.001	0.521

	Counts portion of the model, AV = Crim				Rate ratio

Intercept	1.237	0.087	14.191	<0.001	3.445
zFPP	0.219	0.063	3.455^∗∗^	<0.001	1.245

*FPP (predictor) is z-standardized.*

*Crim = Frequency of self-reported criminality, Crim#0 = Criminality is 0.*

*^∗∗^*p* < 0.01.*

#### Criterion Validity for Inmates

To examine criterion validity for inmates, Spearman rank correlations with variables of (1) current offense, (2) criminal history, and (3) misconduct in prison are reported in **Table 7**. FPP is not associated with length of sentence but its score is higher when index offense is bodily harm or extortion. The FPP correlates significantly with the age of first offense/first violent offense and with the number of violent convictions but not with the number of general convictions. Educational and disciplinary measures are highly associated with overall FPP scores, regardless of which kind of misconduct caused these measures.

**Table 7:** Spearman rank correlations r of FPP and criteria in a sample of inmates (*n* from 109 to 171).

Variables of current offense	FPP
(1) Length of sentence	–0.094
(2) Index offense is bodily harm	0.250^∗∗^
(3) Index offense is fraud	0.073
(4) Index offense is violence	0.110
(5) Index offense is extortion	0.213^∗∗^

**Variables of criminal history**	**FPP**
(6) Number of crimes	–0.049
(7) Age of first offense	–0.227^∗∗^
(8) Age of first violent offense	–0.236^∗^
(9) Number of violent offenses	0.206^∗∗^
(10) Number of probation revocation	0.178^∗^

**Variables of misconduct in prison**	**FPP**
(11) Educational and disciplinary measures	0.315^∗∗^
(12) Educational and disciplinary measures due to violence	0.187^∗^
(13) Educational and disciplinary measures due to insult	0.213^∗∗^
(14) Educational and disciplinary measures due to rule violation	0.204^∗∗^
(15) Forms of parole	0.040

*^∗^*p* < 0.05; ^∗∗^*p* < 0.01.*

#### Cross Validation with Online Sample

To cross validate the six-factor structure in an independent sample, we conducted a CFA in a second study based on *N* = 513 participants from the civil population. Model fit of the six-factor model with intercorrelated factors (Model 1) was considered mostly acceptable and comparable to the CFA results of the main sample with χ^2^(390) = 986, *p* < 0.001; χ^2^*/d*f = 2.5; *RMSEA* = 0.054 [0.05–0.059]; *CFI* = 0.83; *SRMR* = 0.065. However, the *CFI* again falls below the recommended threshold.

## Discussion

The present study deals with the development and validation of the Questionnaire of Psychopathic Personality Traits (FPP). This new questionnaire is based on the PPI (Lilienfeld and Andrews, 1996), which has certain shortcomings although being the best established and well-validated self-report instrument to assess psychopathic traits.

Overall results indicated that the new questionnaire shows an improvement concerning the five points in comparison to the PPI. It is more economic with 30 items compared to 154 items of the PPI-R but nevertheless shows comparable psychometric properties (e.g., reliability, validity). Furthermore, a high convergent correlation between PPI and FPP indicates that the latter captures much of the information that is represented in the former, with fewer items. Item wording should be easy to understand and independently rated from education. A comparison between the civil and offender sample with regard to item characteristics revealed that most of the items showed the same characteristics within both samples. This allows for adequate interpretation of differences in all available scales. Items were intended to have medium situation specificity. Although there is no statistical index that reveal the degree of item specificity, item discrimination indices as well as factor loadings revealed that psychometric properties of most items were satisfying. Results indicate that, in accordance with development requirements, the FPP is independent of positive and negative affectivity. Furthermore, the non-significant correlation with *Unlikely Virtues Scale* suggests that the FPP is not affected by aberrant response styles. Our last two points of criticism concerned the facets of psychopathy and the corresponding factor structure of the instrument. In the first step, we postulated a model of psychopathy and its facets based on theoretical as well as empirical findings of scientific literature to not rely solely on correlation analyses. This allows for testing theoretically derived hypotheses to validate the instrument. Examination of FPPs factor structure as well as psychometric properties was based on a sample that was representative of FPPs field of application and implies the whole range of possible manifestations of psychopathy. Findings revealed that the postulated six-factor structure is represented by the items and scales of the FPP in both subsamples as well as in the total sample. This was additionally supported by the CFA results of the cross validation study.

Besides the five points of criticism further results support good psychometric properties as well as high validity of the FPP. Conducting confirmatory analyses, model fit indices indicated acceptable model fit in the development as well as in the cross-validation sample, besides CFI which did indicate poor model fit. High discrepancy between descriptive measures of overall fit (e.g., RMSEA) and CFI might occur due to low item-intercorrelations in the analyzed sample. Since the CFI implies a ratio between the target model and an independence model, where all variables are assumed to be uncorrelated, low item-intercorrelations might lead to a small difference between both models leading to a lower CFI. Nevertheless, confirmatory analyses demonstrated for both, the development sample and the cross validation sample that the postulated six-factor structure can be accepted for the FPP and subscales represent valid dimensions of the questionnaire.

Analyses of items revealed good indices with respect to item difficulties, item discriminations, and item factor loadings. Reliability of the total psychopathy score was found to be satisfying, reliability for the subscales was satisfying as well for *Lack of Empathy, Fearlessness, Impulsivity*, and *Social Manipulation* but not for *Narcissistic Egocentrism* and *Power*. These results might be attributed to the low number of items per scale. If a scale implies just a small number of items, these items should exhibit different contents to maintain content validity, leading to small intercorrelations between items. Since, measures of internal consistency are sensitive to the number of items and to their intercorrelations (Cortina, 1993) other indices should be taken into account to estimate reliability (Guilford, 1954). Test–retest estimation in Schüller (2017) revealed retest coefficients of *Narcissistic Egocentricity* (*r_tt_* = 0.70) and *Power* (*r_tt_* = 0.86). Thus, internal consistency is acceptable in most scales but not in *Narcissistic Egocentricity* and *Power*, which displayed acceptable test–retest reliability in another study.

Multigroup analysis was conducted to test whether measurement characteristics of the FPP differ in samples of offenders versus civil participants. Results supported configural and metric invariance but full scalar invariance could not be assumed for the FPP. This indicates that inmates agreed more easily to six FPP items without exhibiting higher psychopathic traits. Possible reasons could be the highly structured daily routines in prison or social norms that might arise in closed systems as prisons. Although these six items differ in intercepts, partial scalar invariance was accepted to be a sufficient condition to interpret scale differences in both subgroups (Byrne et al., 1989).

Construct validation analyses indicated a pattern of high convergence between FPP scales and PPI scales. Subscales showed significant and mostly highest correlation indices with theoretically associated subscales of the PPI. However, *Impulsivity* (FPP) showed the smallest correlation with other subscales of the PPI. Theories on subtypes of psychopathy postulate impulsivity to be indicator of the second, antisocial type of psychopathy (Karpman, 1948). Lower correlations of impulsivity with other scales could stand for its strong belonging to the second subtype.

Construct validity of FPP is further reflected by an association with narcissistic, antisocial, little self-critical, and little helpful personality style. Only the lack of association between FPP and *Focus on norms and control* and its positive association with *Attachment* are contrary to our expectations. The zero correlation between FPP and *Focus on norms and control* could be due to competing associations with FPP subscales. High *Focus on norms and control* goes along with high *Empathy* (which is low psychopathy) in order to avoid hurting anyone’s feelings whereas high *Power* and *Social Manipulation* (which is high psychopathy) need some systematic planning and adherence to social norms. These opposite effects might lead to the observed zero correlation. The unexpected positive association between *Attachment* and the FPP might be attributed to the lack of differentiation between attachment styles in this operationalization. Attachment in this study is defined as closeness of a relationship between the participant and his or her (ex-) partner which is found in the dysfunctional ambivalent attachment style. Thus, psychopathic traits such as *Narcissistic Egocentrism, Power*, and *Social Manipulation* might profit of close relationships although they are not necessarily secure and healthy.

Psychopathy has always been associated with antisocial or criminal behaviors or both (Cleckley, 1941; Lykken, 1995; Hemphill et al., 1998; Lynam and Miller, 2012). Mean differences between the samples revealed that inmates had significantly higher values on three of six subscales, *Lack of Empathy, Fearlessness*, and *Impulsivity*, whereas the other three subscales, *Narcissistic Egocentrism, Social Manipulation*, and *Power*, did not differ between inmates and civilians. Since not all facets of psychopathy are highly associated with criminality (Miller and Lynam, 2011; Lilienfeld et al., 2012; Lynam and Miller, 2012) the former three subscales might reflect aspects of psychopathy that are associated with direct criminal behavior while the latter three, *Narcissistic Egocentrism, Social Manipulation*, and *Power* might represent facets of psychopathy that are found in adaptive psychopaths. These differences remained significant when age, sex, and education differences were controlled for, supporting attribution of differences to psychopathic traits.

The FPP predicted self-reported antisocial behavior very precisely in both samples. Regarding the prediction of criminal behavior, the FPP turned out to be better in predicting whether someone committed crimes in general, than in predicting the number of criminal acts that were reported, if any. Assessing self-reported antisocial and criminal behavior carries the advantage of capturing behavior that is not officially recorded and is otherwise part of the dark figure of crime. Disadvantages of self-report might lie in a measurement error due to false memories and an overestimation of the association with the FPP, since both instruments rely on self-reports and share the same method variance.

In the third part of criterion validity, we revealed that the FPP was not associated with length of sentence. Concerning this result Heinzen et al. (2011) found that the Psychopathy Checklist Screening Version (PCL:SV, Hart et al., 1995) Factor 1 (Interpersonal Features) was positively related to the length of conviction, whereas Factor 2 (Social Deviance) was negatively associated with this criterion. Without differentiation of subfactors, no association would be revealed, which might be attributed to construct characteristics rather than to assessment properties. Unlike our hypotheses the FPP showed no association with the number of offenses in an individual’s criminal history, but when divided into different types of conduct, according to our hypotheses the number of violent offenses and probation revocation as well as the age at first general and violent offense showed small but significant associations with FPP. An analysis of FPP and inmates’ misconduct revealed that FPP scores correlated with the number of educational and disciplinary measures due to violence, insults, and rule violation. The degree of parole showed no association with the FPP score. Overall, the correlation pattern underscores associations with violent, impulsive misconduct with small effect sizes.

### Limitations

Despite encouraging results for the FPP, the present study has some limitations. The two subsamples were sampled with different strategies, which could lead to undesired selection effects. While all participants in the sample of offenders were paid for their participation, civil participants were rewarded with the opportunity to participate on a game lottery. Civil participants were recruited in the environment of the research team thus leading to further selection effects regarding some personality traits such as conscientiousness and agreeableness.

The two subsamples differ regarding education, sex, and age. Due to this, the effects of incarceration and education are confounded. However, both variables could be hard to separate. Block formation is very difficult because these variables are highly correlated and cannot be separated in the field, so that highly educated inmates and uneducated civilians may be difficult to find due to low base rates. Furthermore, consideration of education as a control variable may lead to a leveling of effects. Although we already provided first promising results within the online sample, all other results need to be cross-validated with further samples drawn from the civil as well as from the offender population. Additionally, future studies need to be conducted to further examine unexpected findings (e.g., positive correlation with attachment, no correlation with focus on norms and control) in a more elaborate way.

## Conclusion

The FPP is an instrument that allows for a reliable and valid measurement of psychopathic personality traits. The first field of application of the FPP are research studies, where samples of civil participants and inmates have to be compared, because the item wording is suitable for administration in prison contexts, and partial invariance allows for valid interpretation of mean differences. Furthermore, based on its high economy it can be easily integrated in large-scale studies. The second field of application might be practical settings, where the FPP could be used as screening device, yet with some limitations. Since the scales *Narcissistic Egocentrism* and *Power* have shown low internal consistency, these subscales have to be interpreted with caution. However, the FPP should not be applied for risk assessment, because this study does not provide any result that indicates an association between FPP scores and recidivism. Nevertheless, associations with educational and disciplinary measures suggest the benefit of application in correctional facilities and help to plan the process of enforcement of conviction and therapeutic interventions.

## Author Contributions

SE substantially contributed to the conception and design of the work, interpreted the data, wrote the manuscript, and revised it critically for important intellectual content. SR supervised the design of the study and data collection and contributed to data analysis as well as writing and revising the manuscript. All authors contributed to and approved this final version of the manuscript.

## Conflict of Interest Statement

The authors declare that the research was conducted in the absence of any commercial or financial relationships that could be construed as a potential conflict of interest.

## Footnotes

1 Many statements in this paper refer to both versions, the original PPI and its revised version PPI-R. To keep things simple, the term “PPI” is used for both editions.

2 These correlations are based on the civilian sample.
